# Upregulated beta-defensin-1 in murine and human biliary atresia associates with human native liver survival

**DOI:** 10.1038/s41598-026-43602-9

**Published:** 2026-03-26

**Authors:** Christoph Slavetinsky, Jule Basenach, Pascal Damm, Caterina Bertolini, Maximilian Holweg, Steffen Hartleif, Johannes Hilberath, Stephan Singer, Claus Petersen, Jörg Fuchs, Ekkehard Sturm

**Affiliations:** 1 Department of Pediatric Surgery and Urology, University Children’s Hospital Tübingen, Hoppe-Seyler-Straße 3, 72076 Tübingen, Germany; 2 Cluster of Excellence EXC 2124 Controlling Microbes to Fight Infections, Tübingen, Germany; 3 Pediatric Gastroenterology and Hepatology, University Children’s Hospital Tübingen, Tübingen, Germany; 4 Department of Internal Medicine I, University Hospital Tübingen, Tübingen, Germany; 5 Department of Pathology, University Hospital Tübingen, Tübingen, Germany; 6 Pediatric Surgery, Hannover Medical School, Hannover, Germany

**Keywords:** Biliary atresia, Defensins, hBD1, Pediatric cholestatic diseases, Diseases, Gastroenterology, Medical research

## Abstract

Biliary atresia (BA) is a neonatal cholangiopathy that often progresses to cirrhosis despite timely Kasai portoenterostomy (KPE), and prognostic biomarkers remain undefined. Given its role in adult cholestasis, we evaluated human beta-defensin-1 (hBD1) in murine and human BA for associations with disease progression and outcome. This study analyzed hepatic expression of *hBD1* and *TGF-ß* by qRT-PCR in BA at KPE (*n* = 36) and liver transplantation (LT, *n* = 44), and compared with normal (*n* = 15) and cholestatic disease controls (*n* = 36). Serum hBD1 was measured by ELISA in BA (*n* = 23) and healthy infants (*n* = 11). Murine *BD1* was assessed in a Rhesus rotavirus (RRV) BA model. *BD1* was found to be upregulated in murine and human BA, with higher expression at LT than at KPE. Hepatic *hBD1* correlated with *TGF-ß* (R^2^ = 0.21), Ishak fibrosis score (R^2^ = 0.36), and serum bile acids (R^2^ = 0.23). Serum hBD1 was elevated in BA versus controls. Elevated hBD1 at KPE predicted persistent jaundice and reduced native liver survival (X^2^ = 9.5), with ROC analysis showing good discrimination for failure of jaundice clearance at 3 months post-KPE (AUC 0.81 for liver and 0.87 for serum). Thus, hBD1 may serve as a negative predictor of jaundice clearance and native liver survival at the time of KPE.

**Supplementary Information:**

The online version contains supplementary material available at 10.1038/s41598-026-43602-9.

## Introduction

Biliary atresia (BA) is characterized by a progressive, inflammatory and fibro-obliterative obstruction of bile ducts. Despite an unclear pathogenesis, etiological studies have focused on infection-triggered immune dysregulation in the liver and bile ducts^1–3^. However, gene dysregulation, ciliary dysfunction, redox stress, hypoxia and environmental insults were recently suggested to play a role^4,5^. When untreated, BA leads to rapid liver fibrosis, portal hypertension and subsequent liver failure^1^. The first surgical treatment aims at restoring bile flow via excision of the obliterated extrahepatic bile ducts and anastomosis of a jejunal Roux loop with the denuded and transected portal plate, in the so-called Kasai portoenterostomy (KPE). This enables long-term survival with the native liver in 20–40% of patients. Nevertheless, approximately 50% of all patients require liver transplantation in the first 2 years of life and approximately 60–80% of patients in later childhood, adolescence or adulthood. Thus, BA remains major indication for pediatric liver transplantation^1^. Despite being a life-saving procedure, transplantation carries a high risk for surgical complications and exposes the patient to life-long immunosuppression affecting morbidity (e.g., post-transplant lymphoproliferative disease, arterial hypertension, decreased quality of life) and mortality^6,7^. Predicting outcome of biliary atresia after KPE remains challenging. KPE performed at an age < 45 days was associated with a better outcome and is the only established predictor at KPE so far^8,9^. Both normalization of serum bilirubin (clearance of jaundice) and normalization of serum bile acids (additional to normalization of bilirubin) at 3- or 6-months post KPE has been identified as predictor of native liver survival^10^.

Antimicrobial peptides (AMP) such as human beta-defensin-1 (hBD1) belong to the first line of innate immunity against invading microbes. However, they have gained increasing attention due to their roles in immune modulation and as biomarkers. Defensins fulfil important functions in wound healing, promoting matrix metalloproteinase activity, re-epithelization, angiogenesis and fibroblast activation^11–13^. Of note, prolonged high levels of hyaluronic acid, typical for fetal wound healing, have been observed around the duct structures in BA and have been implicated in pathognomonic bile duct obstruction and subsequent liver failure^5^. *hBD1* is expressed in epithelial cells, leukocytes, hepatocytes and the biliary tree and is released from mucosal surfaces^14–16^. It has been found to be upregulated in adult chronic liver disease and to be a highly predictive serum marker of mortality in patients with acute-on-chronic liver failure^15,17^.

The hepatic expression and function of AMPs such as hBD1 in healthy and diseased liver in pediatric cohorts are limited. We aimed to characterize the expression of *hBD1* in BA compared to normal and cholestatic disease controls and to associate expression patterns with clinical course and outcome after KPE. To complement the human analyses, we examined mouse beta-defensin-1 (mBD1) in the RRV neonatal mouse model, a widely used experimental model of BA^18,19^. Since *hBD1* can be induced by inflammatory stimuli and bile-associated factors, we also explored associations with cholestasis and fibrotic activity^15,20^. Here we show that the levels of *hBD1* may be a suitable outcome predictor at the time of KPE and may contribute to the risk stratification of children undergoing KPE for BA.

## Results

### Beta-defensin-1 is expressed in murine liver and is upregulated in experimental biliary atresia

**Fig. 1:**
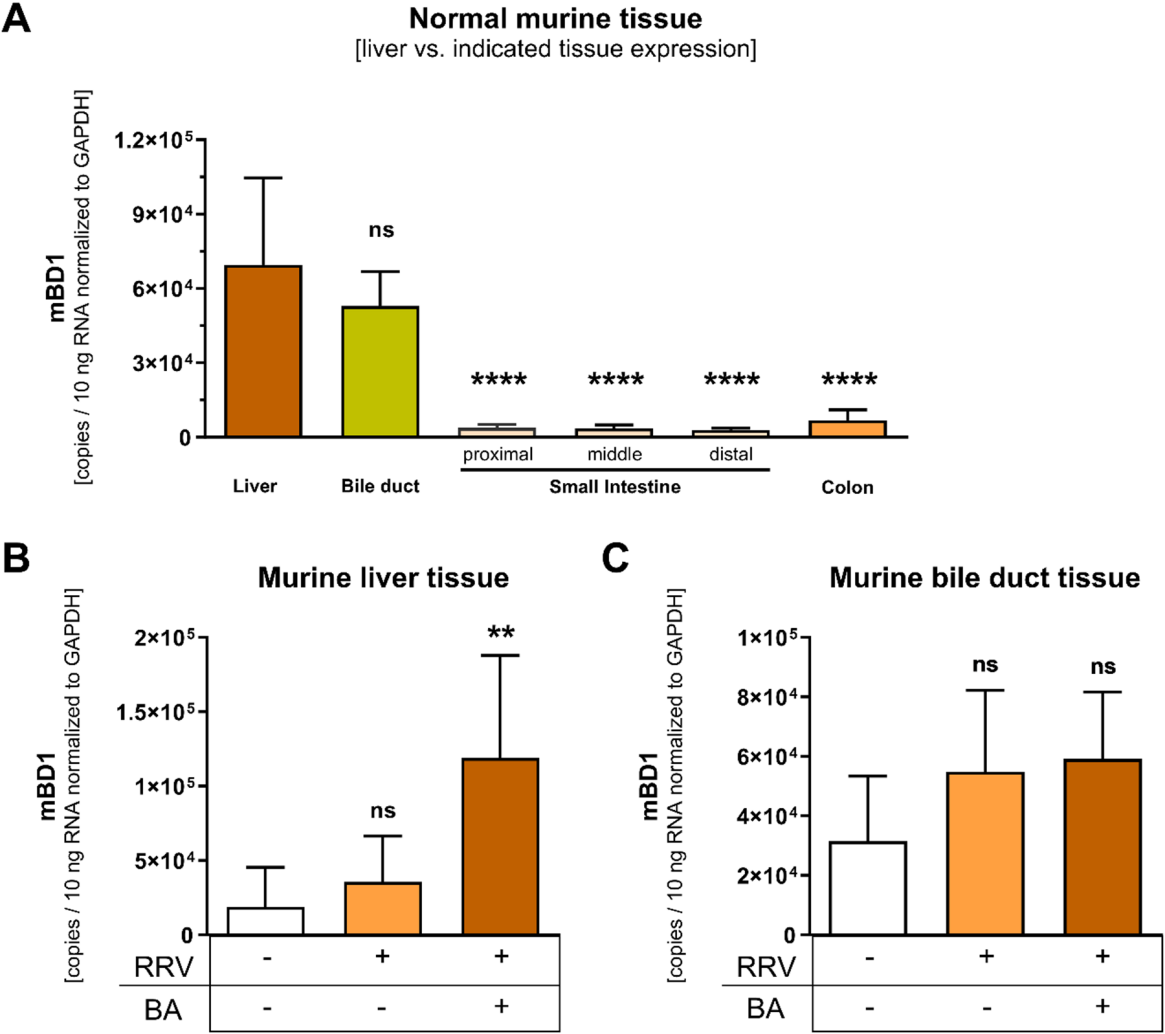
Expression of mouse beta-defensin-1 (*mBD1*) in the murine experimental biliary atresia. (**A**) Expression (copies/10 ng RNA normalized to *GAPDH*) of *mBD1* in the normal neonatal murine liver compared to intestinal and bile duct tissue as measured by qRT-PCR (*n* = 24 mice, normal mouse cohort). Significance symbols indicate pairwise comparisons of liver vs. each indicated tissue based on Tukey-adjusted *p* values. (**B**) Liver expression of *mBD1* in an RRV model of experimental BA in Balb/c compared to mice that did not receive i.p. RRV injection and did not develop BA (*n* = 4), mice receiving RRV injection but not developing BA (*n* = 10), and mice receiving an RRV injection and developing BA (*n* = 10). Significance symbols indicate pairwise comparisons of RRV receiving mice groups versus non-RRV mice. (**C**) Bile duct expression of *mBD1* in the mouse cohort from (**B**). Significance symbols indicate pairwise comparisons of RRV receiving mice groups versus non-RRV mice. Means and 95% confidence intervals are shown. Group differences were analyzed by one-way ANOVA with Tukey’s multiple comparisons test (ns, *p* > 0.05 ; *, *p* ≤ 0.05; ****, *p* ≤ 0.0001). Abbreviations: BA, biliary atresia; RRV, Rhesus Rotavirus; RT-PCR, real-time polymerase chain reaction.

We assessed expression of the murine *hBD1* homolog *mBD1* in liver and intestinal tissues from the RRV neonatal mouse model of experimental BA. In normal mice, *mBD1* was highly expressed in neonatal liver and bile duct tissue (Fig. 1A). *mBD1* expression in murine liver and bile duct was significantly higher than in intestinal tissue (Fig. 1A). To analyze whether *mBD1* expression differs in murine liver disease vs. controls, we compared hepatic gene expression in animals without and with experimentally induced BA. Expression of *mBD1* in the liver was significantly upregulated in mice that developed BA compared to both non-infected mice and mice that received RRV but did not develop BA (Fig. 1B). Of note, *mBD1* upregulation was restricted to liver tissue as there was no significant upregulation of *mBD1* in (extrahepatic) bile ducts after induction of experimental biliary atresia (Fig. 1C).

#### BD1 is upregulated in liver and serum in human biliary atresia and its expression increases during disease progression

To assess *hBD1* expression in human biliary atresia, we analyzed liver biopsy material from patients with biliary atresia at an early-stage (at KPE; eBA) and a late-stage in disease progression (at liver transplantation; lBA) (Table 1). In comparison, we analyzed material from age-matched normal and disease controls (Progressive Familial Intrahepatic Cholestasis (PFIC) and Alagille Syndrome (ALGS)) both at early (eFCD) and late (lFCD) disease stage (Table 1). Group differences in key laboratory parameters are summarized in Table 1 (overall *p* values) and Table S1 (post-hoc comparisons).

**Table 1:** Patient characteristics.

Variable	Normal controls	TNC patients	BA patients		FCD patients		Overall *p* value
			eBA	lBA	eFCD	lFCD	
Number of samples	15	12	83		24		–
			39	44	18	6	–
Male, n of total	11/15	9/12	45/83		14/24		–
			21/39	24/44	12/18	2/6	–
Non-syndromic BA	–	–	34/39	41/44	–	–	–
Syndromic BA	–	–	5/39	3/44	–	–	–
ALGS	–	–	–	–	3/18	2/6	–
PFIC	–	–	–	–	15/18	4/6	–
Age at KPE or sample, d	560 (330–883)	48 (35–66)	51 (36–84)	213 (160–835)	804 (164–1817)	1404 (334–3533)	< 0.0001
Total bilirubin at biopsy, mg/dl	–*	8.0 (5.8–8.9)	9.9 (6.7–12.3)	6.9 (1.6–20.9)	5.8 (0.8–11.7)	17.3 (1.7–23.6)	< 0.0001
ALT at biopsy, U/l	–*	146 (83–225)	210 (154–254)	181 (89–466)	86 (60–499)	211 (75–229)	< 0.0001
AST at biopsy, U/l	–*	85 (48–128)	153 (101–249)	113 (51–257)	75 (30–236)	81 (58–101)	< 0.0001
GGT at biopsy, U/l	–*	176 (81–642)	439 (238–627)	152 (71–346)	43 (20–138)	197 (48–256)	< 0.0001

Data are shown as frequencies or as medians with interquartile range. Overall *p* values were calculated using Kruskal-Wallis tests and are shown in the rightmost column; Dunn’s post-hoc comparisons are provided in Table S1. Abbreviations: ALGS, Alagille syndrome; ALT, alanine transaminase; AST, aspartate transaminase; BA, biliary atresia; eBA, early biliary atresia; eFCD, early familial cholestasis disease (PFIC or ALGS); FCD, familial cholestasis disease (PFIC or ALGS); GGT, gamma-glutamyl transferase; KPE, Kasai portoenterostomy; lBA, late biliary atresia; lFCD, late familial cholestasis disease (PFIC or ALGS); TNC, transient neonatal cholestasis.

*An increase in bilirubin or liver enzymes was an exclusion criterion for normal controls.

**Fig. 2:**
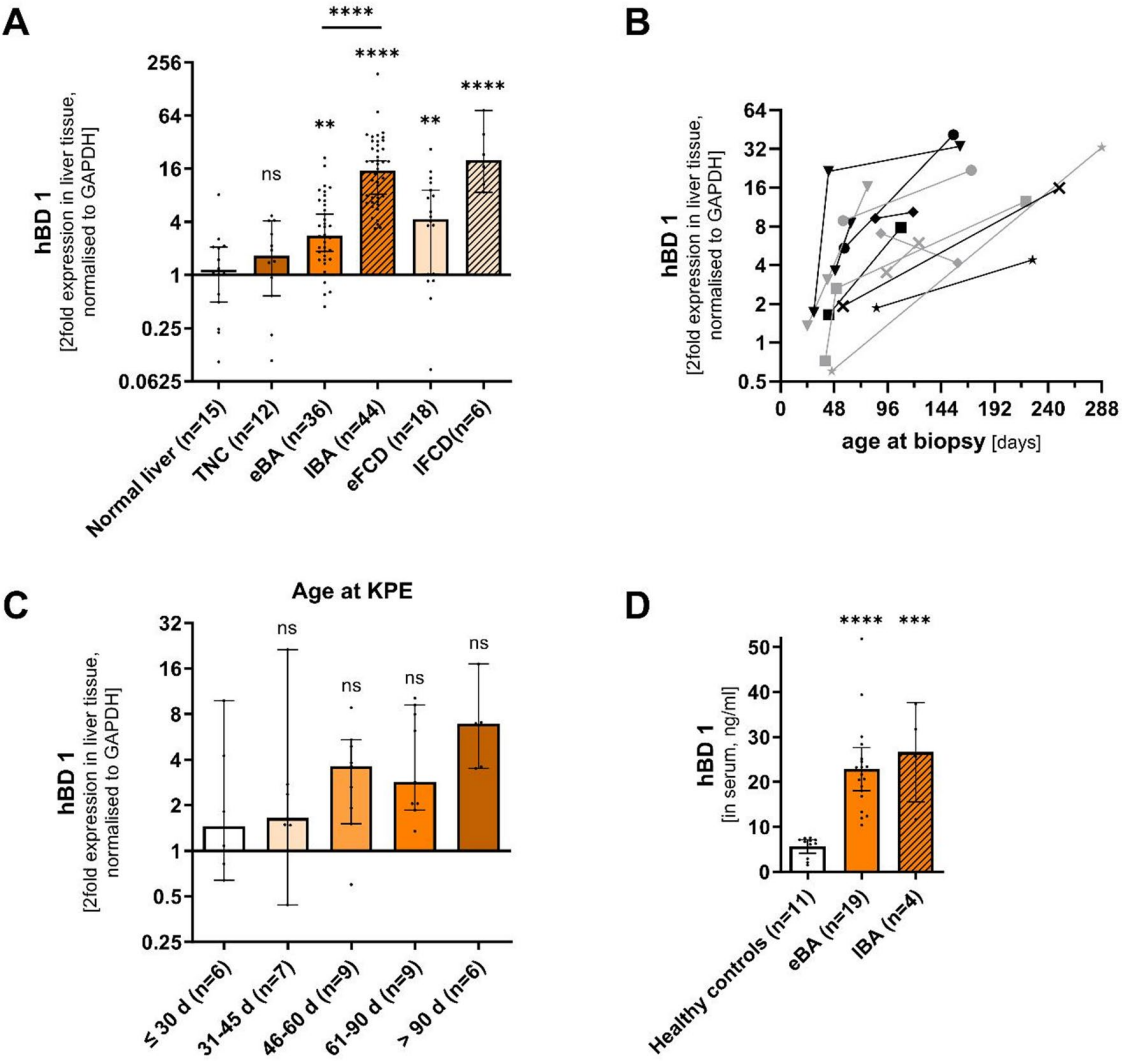
Liver and serum hBD1 in biliary atresia. (**A**) Liver expression (2-fold expression normalized to *GAPDH*) of *hBD1* in normal liver compared to TNC, eBA, lBA, eFCD and lFCD as measured by quantitative RT-PCR. Expression in eBA was further compared to lBA. (**B**) Expression dynamics of *hBD1* (2-fold expression normalized to *GAPDH*) in serial liver biopsies during BA progression as a function of age at biopsy. The different point shapes represent results from individual BA patients. (**C**) Liver expression (2-fold expression normalized to *GAPDH*) of *hBD1* in eBA grouped after age at KPE as indicated in days. (**D**) Serum levels of hBD1 in healthy age-matched individuals compared to eBA and lBA measured by ELISA.Means and 95% confidence intervals are shown. Group differences were analyzed by one-way ANOVA (ns, *p* > 0.05 ; **, *p* ≤ 0.01; ***, *p* ≤ 0.001; ****, *p* ≤ 0.0001). Abbreviations: eBA, early biliary atresia; eFCD, early familial cholestasis disease; KPE, Kasai portoenterostomy; lBA, late biliary atresia; lFCD, late familial cholestasis disease; TNC, transient neonatal cholestasis.

**Fig. 3:**
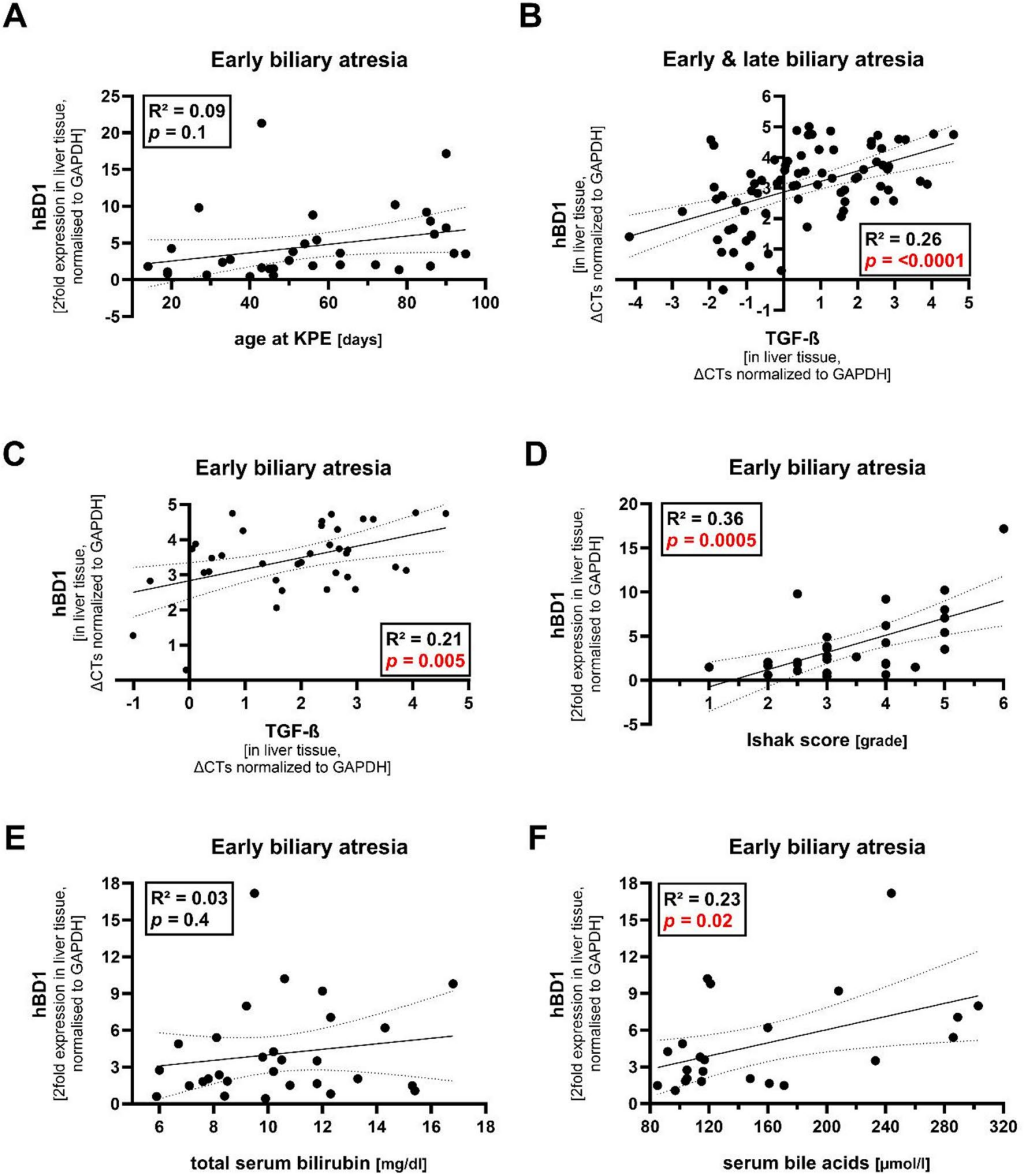
Correlation of *hBD1* liver expression with markers of liver fibrosis and cholestasis. (**A**) Linear regression analysis of liver expression of *hBD1* and age at KPE in days in eBA. Two-fold *hBD1* levels normalized to *GAPDH* versus patient age at KPE corresponding to biopsy date are indicated. (**B**) Linear regression analysis of liver expression of *hBD1* and *TGF-ß* in eBA and lBA combined. ∆CTs normalized to *GAPDH* are indicated. (**C**) Linear regression analysis of liver expression of *hBD1* and *TGF-ß* in eBA. ∆CTs normalized to *GAPDH* are indicated. (**D**) Linear regression analysis of liver expression of *hBD1* and Ishak liver fibrosis score in eBA. Two-fold *hBD1* levels normalized to *GAPDH* versus Ishak score of the respective liver biopsy are indicated. (**E**) Linear regression analysis of liver expression of *hBD1* and total serum bilirubin in eBA. Two-fold *hBD1* levels normalized to *GAPDH* versus total serum bilirubin at biopsy date are indicated. (**F**) Linear regression analysis of liver expression of *hBD1* and serum bile acids in eBA. Two-fold *hBD1* levels normalized to *GAPDH* versus serum bile acids at biopsy date are indicated. R^2^ = and *p* values are shown in each figure, while significant *p* values are marked in red. Abbreviations: eBA, early biliary atresia; KPE, Kasai portoenterostomy; lBA, late biliary atresia.

Expression of *hBD1* in the liver was upregulated in all patients with pediatric cholestatic liver disease irrespective of etiology at early and late disease stages compared to normal controls and benign transient neonatal cholestasis (Fig. 2A). Mean upregulation in eBA was milder than lBA, as some individuals showed an upregulation at KPE (eBA) but others did not (Fig. 2A). Furthermore, expression of *hBD1* in the liver increased during the course of disease, with higher *hBD1* levels in late disease stages (eBA vs. lBA; Fig. 2A) and increasing expression when evaluating individual patients who received serial biopsies (Fig. 2B). Of note, when grouping patients for age at KPE or correlating *hBD1* liver expression with age at KPE, *hBD1* was not significantly increased in older age groups or did not significantly correlate with age at KPE (Figs. 2C and 3A). For comparative purposes, bulk RNA-Seq data from liver samples of patients with BA (at the time of KPE) compared to a heterogeneous group of intrahepatic cholestasis (disease controls) and healthy individuals as controls, were retrieved from the GEO database and analyzed^21^. Consistent with our findings, reanalyzed external transcriptomic data (GEO: GSE46995) showed significantly elevated hepatic *hBD1* expression in BA (Figure S1).

**Fig. 4:**
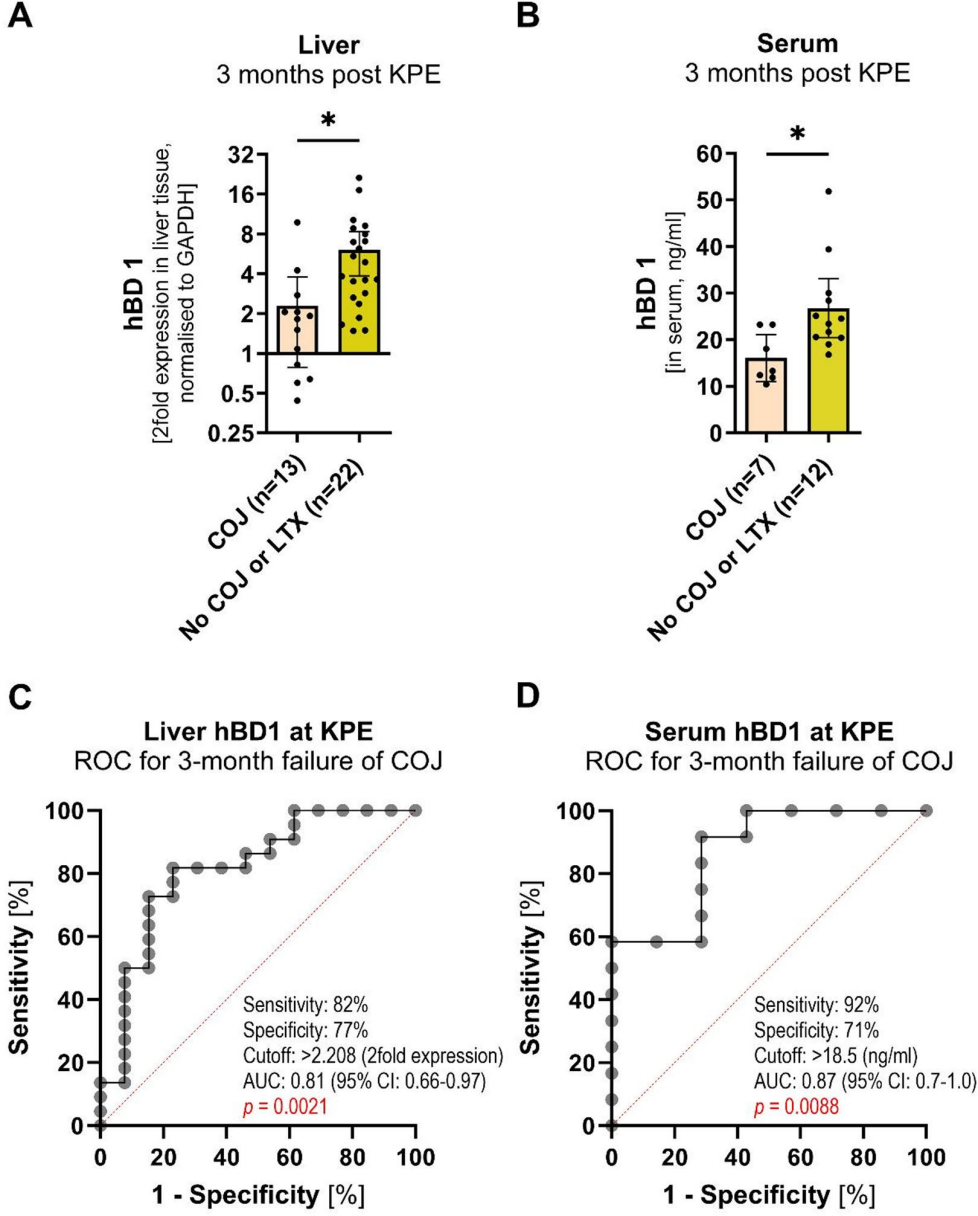
Liver expression and serum level of hBD1 at KPE associates with clearance of jaundice (total bilirubin < 2 mg/dl) 3 months after KPE. (**A**) Liver expression (2-fold expression normalized to *GAPDH*) of *hBD1* in eBA (at KPE) measured by quantitative RT-PCR comparing patients with and without successful clearance of jaundice (COJ) or early liver transplantation (LTX) 3 months after KPE. (**B**) Serum hBD1 in eBA (at KPE) measured by ELISA comparing patients with and without successful clearance of jaundice (COJ) or early liver transplantation (LTX) 3 months after KPE. COJ was defined as total bilirubin < 2 mg/dl 3 months after KPE. (**C**) ROC curve for hepatic hBD1 at KPE predicting 3-month failure of jaundice clearance (event group = no COJ/LTX). (**D**) ROC curve for serum hBD1 at KPE predicting 3-month failure of jaundice clearance (event group = no COJ/ LTX). Means and 95% confidence intervals are shown (**AB**). Group differences were analyzed by t test (*, *p* ≤ 0.05) (**AB**). Abbreviations: COJ, clearance of jaundice; eBA, early biliary atresia; KPE, Kasai portoenterostomy; LTX, liver transplantation; ROC, receiver operating characteristic; AUC, area under the ROC curve; RT-PCR, real-time polymerase chain reaction.

When we compared hBD1 serum levels in biliary atresia vs. healthy, age-matched individuals, we observed significantly increased levels in both early and late stages of biliary atresia compared to healthy children (Fig. 2D). In eBA at the time of KPE, serum hBD1 levels significantly correlated with hepatic *hBD1* expression (Spearman r_s_ = −0.62 for serum hBD1 vs. hepatic ΔCT; Figure S2).

#### Liver expression of hBD1 correlates with serum bile acids and fibrotic activity in biliary atresia

To explore clinical and histological correlates of *hBD1* upregulation in BA, we correlated hepatic *hBD1* expression with serum bilirubin, serum bile acids, C-reactive protein, expression of the profibrotic activity marker *TGF-ß*, and the histological grade of fibrosis (Ishak score). We found that *hBD1* liver expression in early and/or late BA significantly correlated with fibrotic activity (measured by *TGF-ß* liver expression and Ishak fibrosis score) and with serum bile acids (Fig. 3BCDF). There was no correlation between *hBD1* and serum bilirubin or C-reactive protein (Fig. 3E & Figure S3).

#### Human BD1 expression at KPE predicts clearance of jaundice and native liver survival in biliary atresia

**Fig. 5:**
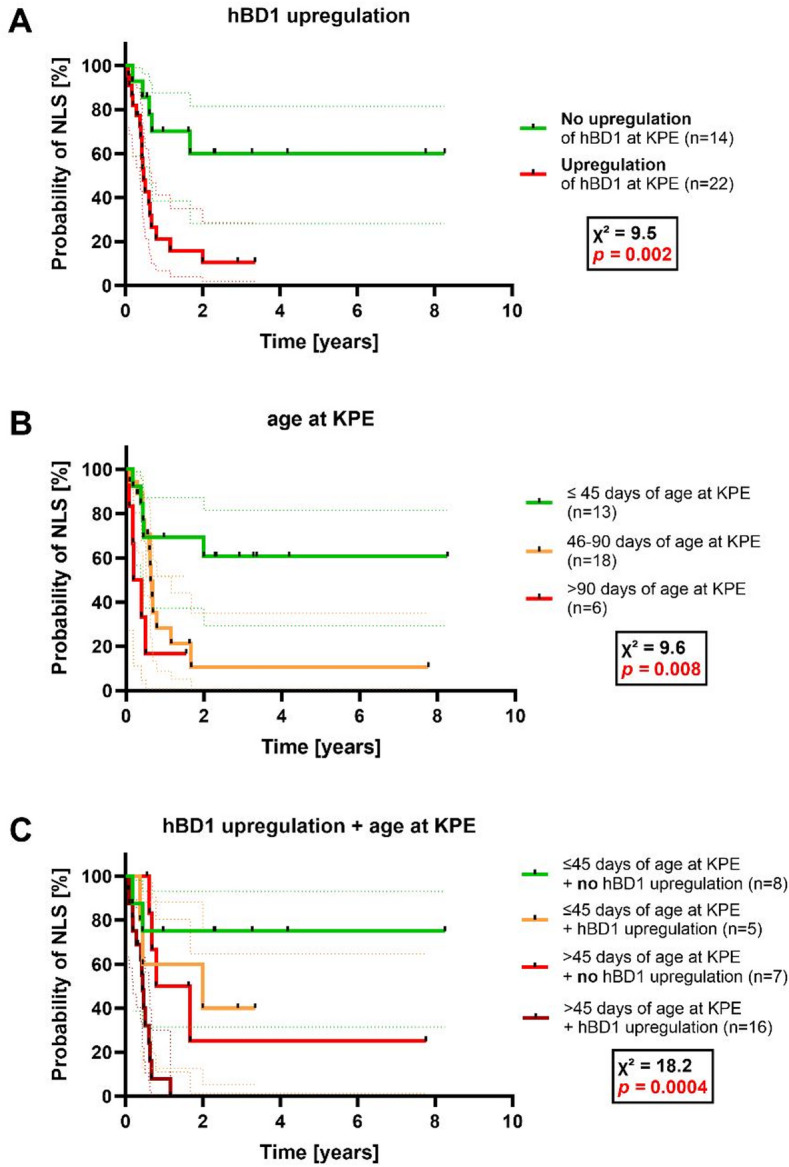
Native liver survival after KPE comparing patients with and without *hBD1* upregulation and grouped by age at KPE. Kaplan-Meier estimator curves for native liver survival at the time of KPE in eBA discriminating (**A**) between eBA patients with or without upregulation of *hBD1* liver expression as measured by RT-PCR (normalized to *GAPDH*), (**B**) between indicated age groups in eBA at the time of KPE, and (**C**) between eBA patients combining presence or absence of *hBD1* upregulation in the liver and ≤ or > 45 days of age at the time of KPE. The dotted lines represent 95% confidence intervals. χ^2^ and *p* values are indicated in each figure, while significant *p* values are marked in red. An upregulation of *hBD1* in eBA was defined as a fold-change above the upper confidence limit of the 95% confidence interval of normal control liver expression. Abbreviations: eBA, early biliary atresia; KPE, Kasai portoenterostomy; NLS, native liver survival.

We assessed whether hBD1 levels at the time of KPE (eBA) were associated with postoperative jaundice clearance and native liver survival. Patients were grouped by clearance of jaundice at 3 months after KPE (defined as total bilirubin < 2 mg/dl) or no clearance of jaundice at 3 months after surgery, with the latter group including patients who received early liver transplantation. Expression levels of *hBD1* in the liver and serum at KPE were significantly increased in patients that failed to clear jaundice 3 months after KPE (Fig. 4AB). Receiver Operating Characteristic (ROC) curve analysis was performed to quantify the discriminatory performance of hBD1 at KPE for failure to clear jaundice at 3 months (event group = non-clearance). For hepatic hBD1, the area under the ROC curve (AUC) was 0.81 (95% CI 0.66–0.97; *p* = 0.0021), with an optimal cutoff of > 2.208-fold expression (Youden index), yielding 82% sensitivity and 77% specificity. For serum hBD1, the AUC was 0.87 (95% CI 0.7–1.0; *p* = 0.0088), with an optimal cutoff of > 18.5 (ng/ml) (Youden index), yielding 92% sensitivity and 71% specificity (Fig. 4CD). Since a failed clearance of jaundice most often requires early liver transplantation, we assessed whether an upregulation of *hBD1* at KPE (defined as fold-change above the upper confidence limit of the 95% confidence interval of normal control liver expression) correlated with shorter native liver survival by Kaplan-Meier-estimator. When *hBD1* was upregulated at KPE, patients showed a significantly shorter duration of native liver survival (survival in approx. 10% of patients after 2 years) compared to patients without upregulation of hBD1 (approx. 65% of patients after 2 years) (Fig. 5A). Of note, *hBD1* liver expression did not correlate with age at KPE (Figs. 2C and 3D). Thus, it is likely to be an independent risk factor. To compare the effectiveness of native liver survival prediction using `age at KPE´, the only established predictor, compared to *hBD1* upregulation, we analyzed our cohort for native liver survival in groups based on age at KPE (≤ 45 days, 46–90 days, and > 90 days of age). As expected, patients with an age of ≤ 45 days at KPE showed a prolonged native liver survival (Fig. 5B). When comparing the predictive power for native liver survival, age at KPE (X^2^ = 9.6) and *hBD1* upregulation (X^2^ = 9.5) were equally predictive. Therefore, we combined both predictors, which allowed an even more accurate prediction of native liver survival (X^2^ = 18.2) (Fig. 5C). Patients ≤ 45 days at KPE with no *hBD1* upregulation survived for 2 years and longer with their native liver in approximately 75% of the cases, while *hBD1* upregulation correlated with a significantly reduced duration of native liver survival in all age groups (Fig. 5C).

## Discussion

Biliary atresia remains a devastating liver disease of infancy with elusive pathogenesis and a rapid disease progression, in the majority of the cases failing to improve long-term after KPE.

The following courses after KPE can be discriminated: (1) patients exhibiting high and persistent levels of serum bilirubin, which reliably predict a poor outcome and the need for early liver transplantation, in many cases in the first year of life^22^, (2) patients achieving normal bilirubin levels but showing a less certain disease course, with 18% needing liver transplantation in the first two years of life and others experiencing portal hypertension as a consequence of advancing liver fibrosis, e.g., 71% of patients developing splenomegaly^23^. 50% of all patients require liver transplantation before adulthood^10,23^. Several studies have investigated biomarkers that could help predict outcome^10,24,25^. However, particularly early in the course of disease as for the time point of KPE, reliable biomarkers are still lacking.

In this study we identify BD1 as a potential biomarker to predict outcome at the time of KPE. Specifically, we show that *BD1* is expressed in pediatric liver tissue and is upregulated in both experimental (mouse) and human BA and in advanced stages of pediatric cholestatic disease controls. Expression of *hBD1* in the liver correlates with serum bile acids and fibrosis markers. An upregulation of hBD1 in serum and liver of patients with BA at KPE is significantly associated with persistent hyperbilirubinemia and shorter native liver survival. Consistent with serum hBD1 reflecting disease-associated hepatic expression, serum hBD1 levels correlated with hepatic hBD1 expression at KPE. These findings suggest that expression of *hBD1* may serve as a predictive biomarker in BA. This prognostic association was supported by ROC analyses for failure of jaundice clearance 3 months post-KPE, showing good discriminatory performance for both hepatic and serum hBD1 at KPE.

In current models of care, managing BA patients in need of liver transplantation is challenging, given a cumulative waitlist mortality of 5.2% in a recent North American study^26^ or 7.9% at 2 years of age in a recent European study^27^. Effective biomarkers predicting surgery outcome at the time of KPE may improve BA care for both patients with long-term native liver survival after KPE and patients requiring organ replacement. Several investigations have analyzed potential outcome predictors in BA. However, so far there is no commonly established biomarker, neither for early diagnosis nor predicting outcome. Serum levels of the matrix metallopeptidase‑7 (MMP‑7) have recently emerged as one of the most promising approaches to support early diagnosis^28^. The age at KPE is still the most established predictor for outcome after KPE^8^. Novel approaches for predictive biomarkers after KPE include the aspartate aminotransferase to platelet ratio index (APRI), serum bile acids, or serum FGF19^10,24,25^. Unfortunately, so far, none of these could be established as a broadly used, reliable biomarker. For our study, it is important to highlight that expression levels of *BD1* were not a marker specific to biliary atresia disease progression. *hBD1* expression is also increased during the progression of other (e.g., hereditary) pediatric cholestatic diseases. Nevertheless, this does not diminish the high predictive value of hBD1 for negative BA outcome and it is desirable to understand how BD1 links to other drivers of BA disease progression.

Recent studies on BA pathogenesis posited an unknown but likely prenatal initial insult, followed by a dysregulated response of neonatal immunity to injury with emerging roles for ciliary dysfunction, redox stress and hypoxia^4^. In particular, an overshooting wound healing program in the fetus, characterized by a hypoxia-induced increase in periluminal deposition of hyaluronic acid in extrahepatic bile ducts, may contribute to BA disease progression^5^. Of note, hyaluronic acid has been shown to induce human beta-defensin-2 in both skin and intestinal epithelium, suggesting a potential association of defensin induction in BA to a prolonged fetal wound healing program^5,29,30^. Furthermore, hBD1 was shown to affect wound healing (e.g. by fibroblast activation) and to possess immunomodulatory functions (e.g. chemoattraction), underlining the potential link to the recently proposed model for BA etiology^12–14,31^. Novel treatment approaches for BA include inhibition of the ileal bile acid transporter (IBAT) with the purpose to reduce the systemic bile acid pool and subsequent toxicity in the liver compartment of BA (e.g., the Phase 3 BOLD study; NCT 04336722). While serum bile acids recently showed potential to predict outcome after KPE in infants achieving bilirubin clearance^10^, studies evaluating the role of bile acids in driving disease in biliary atresia are still lacking. Our data suggest that serum bile acids may trigger *hBD1* upregulation in biliary atresia as we show that serum bile acid levels correlate with *hBD1* expression in individual patients. This is in line with recent in vitro experiments in hepatocyte cell lines showing *hBD1* upregulation upon bile acid trigger^15^. Yet more functional evaluations are needed to confirm this hypothesis. Unexpectedly and strikingly, liver expression of *hBD1* did not significantly correlate with age at KPE. This suggests that hBD1 aligns with the degree of fibrosis, which is often but not always reflected by age at KPE, enabling improved prediction for patients for whom the age at KPE does not correspond to intrahepatic disease status. The role of hBD1 in fibrosis signaling requires further investigation, yet the significant correlation of *hBD1* liver expression and the histological Ishak liver fibrosis score suggests a profibrotic activity of hBD1. It may activate fibroblasts (or stellate cells) as it has been described for defensins in wound healing^13^. Moreover, hBD1 may drive pericanalicular fibrotic activity in the biliary tree in conjunction with deposition of hyaluronic acid as part of an overshooting (fetal) wound healing program.

Despite successfully characterizing the role of BD1 in BA and in pediatric cholestatic diseases, our study has limitations. The proposed cutoffs derived from ROC analyses should be validated in independent, multicenter cohorts before clinical application. Included patient data originate from a single tertiary center in Europe, which, therefore, predominantly represent the Caucasian phenotype of biliary atresia.

We show that an increased *hBD1* expression early in BA is found in patients with less favorable native liver survival, emphasizing hBD1´s potential value as a predictive biomarker. Persistent hyperbilirubinemia 3 months after KPE and shorter native liver survival is found significantly more often in patients with an increased *hBD1* liver expression and serum level at KPE. hBD1 seems to be an autonomous risk factor defining the intrahepatic state of liver disease and fibrotic activity independent of age at KPE, which improves the assessment of BA disease severity. The combination of increased *hBD1* levels with age at KPE even makes prediction of native liver survival more accurate. Early risk stratification with *hBD1* expression as a predictive biomarker at KPE could therefore improve management, especially in patients requiring early transplantation. These could profit from timely identification and preparation for liver replacement reducing the risk for waitlist mortality.

In conclusion, our study identifies BD1 as a potential predictor of outcome in biliary atresia measuring intrahepatic disease progression and fibrotic activity, the likely result of an overshooting wound healing. HBD1 could serve as a predictive biomarker early in pediatric cholestatic diseases (and likely beyond) assessing the risk of liver failure and need for transplantation in BA.

## Methods

### Human liver and serum samples

Liver tissue and corresponding serum samples were collected from our mainly Caucasian patient cohort (Table 1) undergoing a diagnostic percutaneous liver biopsy at the Pediatric Gastroenterology and Hepatology or liver surgery at the Departments of Pediatric Surgery and Urology and General, Visceral and Transplant Surgery at the University Hospital of Tübingen. Surgery indications were BA, intractable cholestatic pruritus, benign and malignant liver masses or secondary biliary cirrhosis. Liver tissue from BA (*n* = 81) was compared to disease controls from transient neonatal cholestasis (TNC) (*n* = 12) and familial cholestatic diseases (FCD) (Alagille syndrome and Progressive Familial Intrahepatic Cholestasis) (*n* = 24). BA and FCD specimens were grouped in early (biopsy at diagnosis, e.g. KPE for BA) or late disease stage (biopsy at liver transplantation). A pathologist examined all tissue samples for entity of tissue, severity of cholestasis, and fibrosis including Ishak scores. Fibrosis was graded using the Ishak scoring system on a 0–6 scale: 0, no fibrosis; 1, fibrous expansion of some portal areas; 2, fibrous expansion of most portal areas; 3, fibrous expansion of most portal areas with occasional portal-to-portal bridging; 4, marked bridging (portal-to-portal and/or portal-to-central); 5, incomplete cirrhosis (bridging with occasional nodules); 6, established cirrhosis^32^. Tissue from normal controls was either histologically normal liver tissue without inflammation or fibrosis resected as surrounding tissue of benign or malignant liver masses (*n* = 11) or healthy, age-matched liver tissue obtained commercially (*n* = 4, Sekisui XenoTech, Kansas City, MO).

Serum samples from healthy, age-matched controls (*n* = 11) were collected and compared to serum samples from BA at early (eBA; at KPE; *n* = 19) and late (lBA; at transplantation; *n* = 4) disease state.

The study was approved by the ethics committees of the medical faculty of the University of Tübingen, Germany and was conducted in accordance with the Declaration of Helsinki (Ref.-No. 230/2021BO2). Written informed consent was obtained from each patient.

### RRV Balb/c mouse model of experimental biliary atresia

Rotavirus-free BALB/c mice were purchased from Charles River (Charles River Laboratories, Research Models and Services, Sulzfeld, Baden-Württemberg, Germany). Pathogen-free laminar-flow cages were used for keeping mice under a 12 h dark-light-cycle. Food, water and litter were sterilized. The animal welfare committee of the Medizinische Hochschule Hannover approved all procedures and analysis in compliance with the national regulations for the protection of animals (permit number 07/1327). This study is reported in accordance with the ARRIVE guidelines. Mice were handled under the supervision of a responsible veterinarian.

To induce biliary atresia in mice, 20-microliter saline solution containing 1 × 10^6^ pfu/mg of Rhesus Rotavirus (RRV; strain MMU18006) or sterile saline in controls were applied intraperitoneally in all newborn mice within 24 h postpartum, as previously described^33^. Death within 48 h after infection was considered as injection-related mortality and dead mice were excluded from the analysis. Animals were regularly monitored for weight, general appearance, and signs of cholestasis such as jaundice.

Mice were sacrificed and dissected on day 5, 8, 11 or 15 after RRV-infection under inhalation anesthesia with isoflurane (3–4% for induction, 1–2% for maintenance in 100% oxygen) delivered via a precision vaporizer and neonatal induction chamber (oxygen flow rate 0.5–1.0 L/min; active waste-gas scavenging). Euthanasia was performed under deep isoflurane anesthesia by cervical dislocation as the physical method, and death was confirmed by sustained absence of respiration and heartbeat under a dissection microscope. The presence of morphological changes in the gall bladder, hepatoduodenal ligament, and corresponding microscopic liver findings was used as a criterion to confirm experimental BA, as previously described^33^. Explanted livers, extrahepatic bile ducts, small intestines (proximal, middle, and distal sections) and colons were divided and harvested for examination. Small samples from each liver were fixed in formalin 4%, embedded in paraffin and stained with hematoxylin and eosin (H.E.). Remaining liver samples were snap-frozen in liquid nitrogen and stored at − 80 °C for virus and mRNA quantification. Approximately 30 mg of liver sample from each mouse was lysed and homogenized using a TissueRuptor and RLT buffer (Qiagen GmbH, Hilden, North Rhine-Westphalia, Germany) following the manufacturer´s instructions of the RNeasy Mini Kit (Qiagen GmbH, Hilden, North Rhine-Westphalia, Germany; see below).

### RNA isolation from liver samples and quantitative RT-PCR to analyze gene expression

For murine expression analysis, RNA was isolated from liver tissue using Trizol/Qiagen RNeasy protocol according to the manufacturer´s instructions as previously described^15^. RNA (1 µg) with an integrity number > 7 was used as a template for cDNA synthesis using the TaqMan Reverse Transcription Kit (Applied Biosystems) according to the manufacturer´s instructions. The levels of *Bd1* mRNA from mouse liver tissue were quantified by LightCycler Real-Time PCR as recently described^34^.

For expression analysis from human liver tissue, RNA was isolated with the Direct-zol™ RNA Miniprep Plus Kit from Zymo Research and according to the manufacturer´s instructions after homogenization of samples using ZR BashingBead Lysis Tubes (2 mm) (Zymo Research Europe GmbH, Freiburg, Germany) in the Precellys Evolution Homogenizer (Bertin Technologies, Montigny-le-Bretonneux, France). RNA concentrations and quality were determined using a NanoDrop™ 2000c spectrometer (Thermo Fisher Scientific, MA, USA).

For the determination of relative amounts of *hBD1*, *TGF-ß*, and *GAPDH* mRNA in the cohort of human liver samples (*n* = 132), the levels of high-quality total RNA were analysed in quadruplicates applying the iTaq Universal One-Step RT-qPCR Kit (Bio-Rad Laboratories, Feldkirchen, Germany) according to the manufacturer´s instructions. Briefly, a reaction mixture was prepared containing RNA template (10 ng), iTaq Universal SYBR^®^ Green Reaction Mix, iScript™ Reverse Transcriptase, 0.5 µM of forward and reverse primer, and nuclease-free water to a final volume of 20 µl. The primers used are listed in Table S2 in the supplementary information. Thermal cycling conditions consisted of an initial reverse transcription step at 50 °C for 10 min, followed by polymerase activation at 95 °C for 1 min, 40 cycles of denaturation at 95 °C for 10 s, and annealing/extension at 60 °C for 30 s. To verify product specificity, a melting curve analysis was conducted from 65 °C to 95 °C, increasing by 0.5 °C per 5 s. All reactions were performed in triplicate using a CFX96 Touch Real-Time PCR Detection System (Bio-Rad Laboratories, Feldkirchen, Germany). Quantification of target gene expression was normalized to GAPDH as internal control, and relative expression changes were calculated using the ΔΔCt method^35^.

### Enzyme-linked immunosorbent assay to quantify hBD1 in serum

Blood samples from participants were collected and allowed to clot for 30 min at room temperature. Serum was separated by centrifugation at 1500 x g for 5 min at 4 °C, aliquoted and stored at -80 °C. Serum levels of hBD1 were quantified using the Human BD-1 ELISA development kit from PeproTech (PeproTech Germany, Hamburg, Germany) and following the manufacturer´s protocol. Briefly, samples were thawed on ice, 100 µl of diluted serum and standards were added in triplicates to a 96-well plate pre-coated with capture antibody, and incubated for 2 h at room temperature. Plates were washed with 0.05% Tween-20 in PBS and incubated with the biotinylated detection antibody followed by incubation with avidin-HRP (horseradish peroxidase). After washing, the ABTS (2,2’-Azino-bis(3-ethylbenzthiazoline-6-sulfonic acid) diammonium salt) substrate was added and the resulting colorimetric reaction was detected with an ELISA plate reader at 405 nm. hBD1 concentrations were determined by interpolation from a standard curve generated from recombinant hBD1 standards with known concentrations.

### Statistical analyses

Gene expression changes are shown as mean fold changes obtained from the ΔΔCT-method with 95% confidence intervals. Group differences and significance of changes were analyzed by one-way ANOVA with Tukey´s multiple comparison test or t-test using GraphPad Prism 10.0.2 software (GraphPad Software, Inc., La Jolla, USA). ΔCT values were used for statistical analysis where applicable.

For clinical data including key laboratory parameters and age at KPE, group differences and significance of changes were analyzed by Kruskal-Wallis test with Dunn´s multiple comparison test using GraphPad Prism 10.0.2 software (GraphPad Software, Inc., La Jolla, USA).

Linear regression analysis was performed to assess the relationship between hBD1 expression and dependent variables using GraphPad Prism 10.0.2 software (GraphPad Software, Inc., La Jolla, USA). The regression model was evaluated by determining the slope, intercept, and goodness of fit (R^2^ value). Assumptions of linearity, normality, and homoscedasticity were verified by examining residual plots and using appropriate diagnostic tests. Statistical significance of the regression coefficients was considered at a *p* value ≤ 0.05. Results are presented with 95% confidence intervals. Correlations were additionally assessed using Spearman’s rank correlation (two-tailed), reporting r_s_ and *p* values.

ROC curve analyses using GraphPad Prism 10.0.2 software (GraphPad Software, Inc., La Jolla, USA) were performed to evaluate hepatic and serum hBD1 at KPE for prediction of failure to clear jaundice at 3 months (event group = non-clearance). AUCs with 95% confidence intervals and *p* values versus AUC = 0.5 were calculated, and optimal cutoffs were determined using the Youden index.

Native liver survival analysis was performed using the Kaplan-Meier estimator assessing time-to-event data using GraphPad Prism 10.0.2 software (GraphPad Software, Inc., La Jolla, USA), with the primary outcome event defined as liver transplantation or death during follow-up. Survival curves were generated, and the median survival times, along with 95% confidence intervals, were calculated with univariable models. The log-rank (Mantel-Cox) test was employed to compare survival distributions between groups. Statistical significance was considered at a *p* value ≤ 0.05. Analyses were performed in compliance with standard practices for survival analysis.

## Supplementary Information

Below is the link to the electronic supplementary material.

Supplementary Material 1

## Data Availability

The datasets generated and analyzed during this study, including human and murine data, are available from the corresponding author upon reasonable request.
