# Supplementary material for: Upregulated beta-defensin-1 in murine and human biliary atresia associates with human native liver survival
Source: Sci Rep. 2026 Mar 26;16:10485. doi: 10.1038/s41598-026-43602-9 (PMC13031917; doi:10.1038/s41598-026-43602-9)
Supplement: Supplementary file 1 — Supplementary Material 1 [file 41598_2026_43602_MOESM1_ESM.docx]

***Supplementary Material***

**Table S1.** Pairwise comparisons of key laboratory parameters and age at KPE across groups from Table 1. *P* values are multiplicity-adjusted post-hoc comparisons following the overall group test (Kruskal-Wallis with Dunn). Normal controls refer to normal liver controls. Abbreviations: ALT, alanine transaminase; AST, aspartate transaminase; eBA, early biliary atresia; eFCD, early familial cholestasis disease (PFIC or ALGS); GGT, gamma-glutamyl transferase; KPE, Kasai portoenterostomy; lBA, late biliary atresia; lFCD, late familial cholestasis disease (PFIC or ALGS).

| Variable | Normal vs. eBA | Normal vs. eFCD | eBA vs. eFCD | lBA vs. lFCD | eBA vs. lBA | eFCD vs. lFCD |
| --- | --- | --- | --- | --- | --- | --- |
| Age at KPE or sample, d | ns | ns | ns | ns | *p*=0.0003 | ns |
| Total bilirubin at biopsy, mg/dl | - | - | ns | ns | ns | *p*=0.01 |
| ALT at biopsy, U/l | - | - | *p*=<0.0001 | ns | ns | ns |
| AST at biopsy, U/l | - | - | *p*=<0.0001 | ns | ns | ns |
| GGT at biopsy, U/l | - | - | *p*=<0.0001 | ns | ns | ns |

**Table S2.** Primers used for quantitative RT-PCR.

| Oligonucleotides for qPCR | Sequence 5`→ 3` |
| --- | --- |
| hBD1 forward | ggcctcaggtggtaactttct |
| hBD1 reverse | ttcttctggtcactcccagc |
| GAPDH forward | gtctcctctgacttcaacagcg |
| GAPDH reverse | accaccctgttgctgtagccaa |
| TGFß forward | gcaagtggacatcaacgggttc |
| TGFß reverse | gtggccatgagaagcaggaaag |
| mBD1 forward | CCAGATGGAGCCAGGTGTTG |
| mBD1 reverse | AGCTGGAGCGGAGACAGAATCC |
| mGAPDH forward | CATCACTGCCACCCAGAAGACTG |
| mGAPDH reverse | ATGCCAGTGAGCTTCCCGTTCAG |


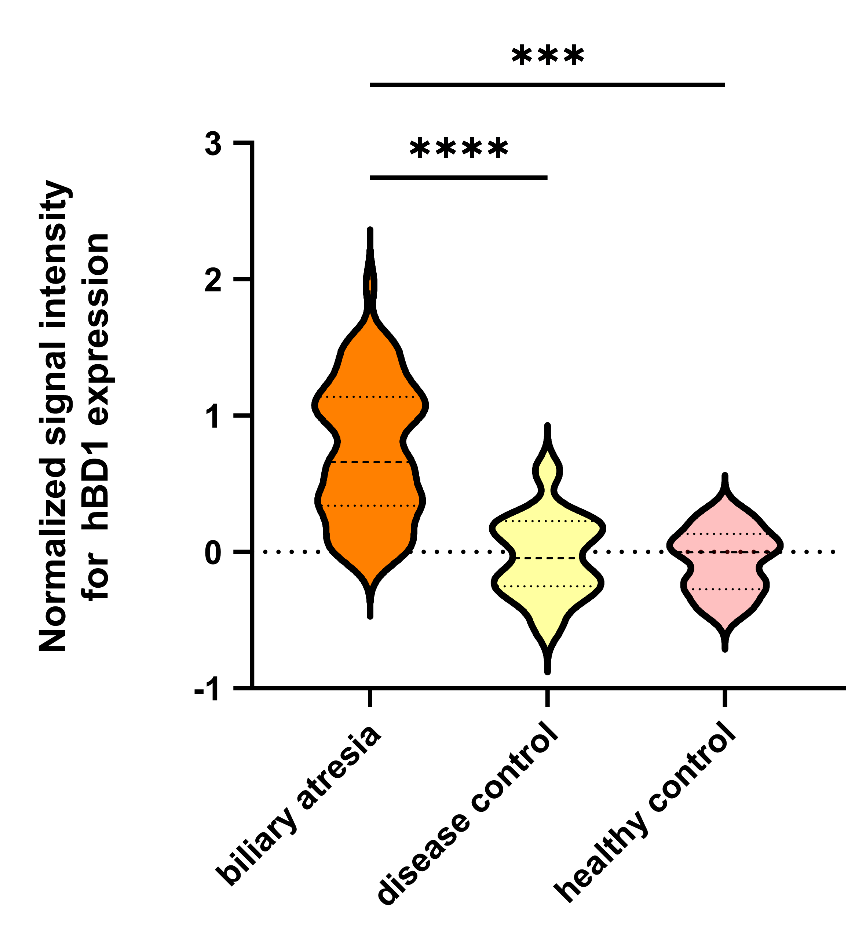


**Figure S1.** External validation dataset: Liver expression of hBD1 (DEFB1) in BA using a published genome-wide expression dataset generated with the GeneChip® Human Gene 1.0 ST Array (1). Expression data from 64 patients with BA at diagnosis, 14 age-appropriate patients with intrahepatic cholestasis as diseased controls, and 7 normal controls were retrieved from the Gene Expression Omnibus [GEO: GSE46995]and reanalyzed.

***
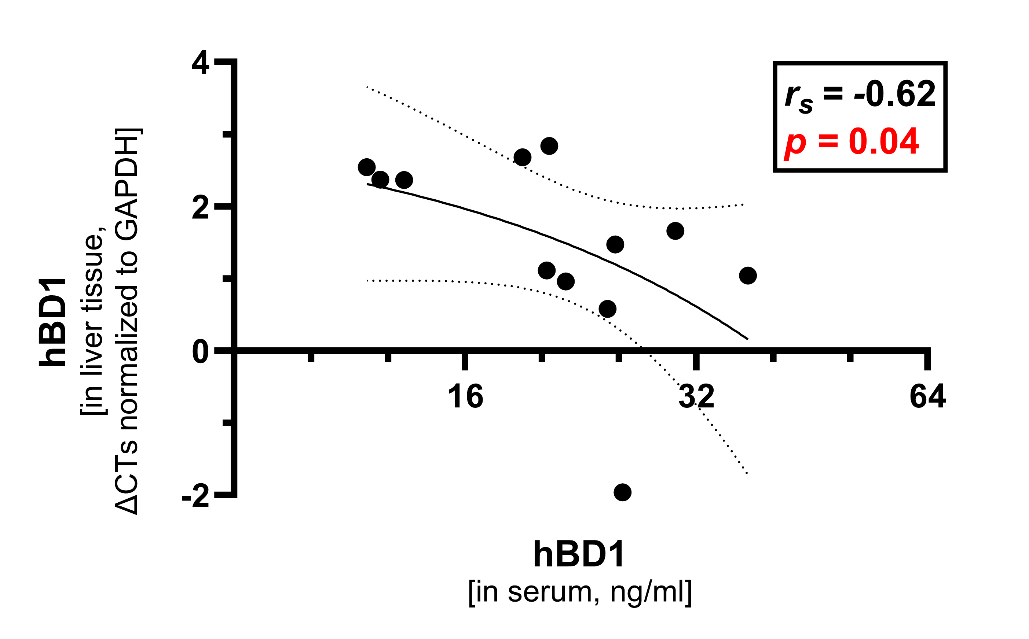
***

**Figure S2.** Correlation of serum and hepatic hBD1 at KPE in eBA. Correlation between serum hBD1 levels (ELISA) and hepatic *hBD1* expression assessed by qRT-PCR and expressed as ΔCT values normalized to *GAPDH* in patients with eBA at KPE. Spearman’s rank correlation was used; r_s_ and *p* value are shown in the figure. Abbreviations: eBA, early biliary atresia; KPE, Kasai portoenterostomy.


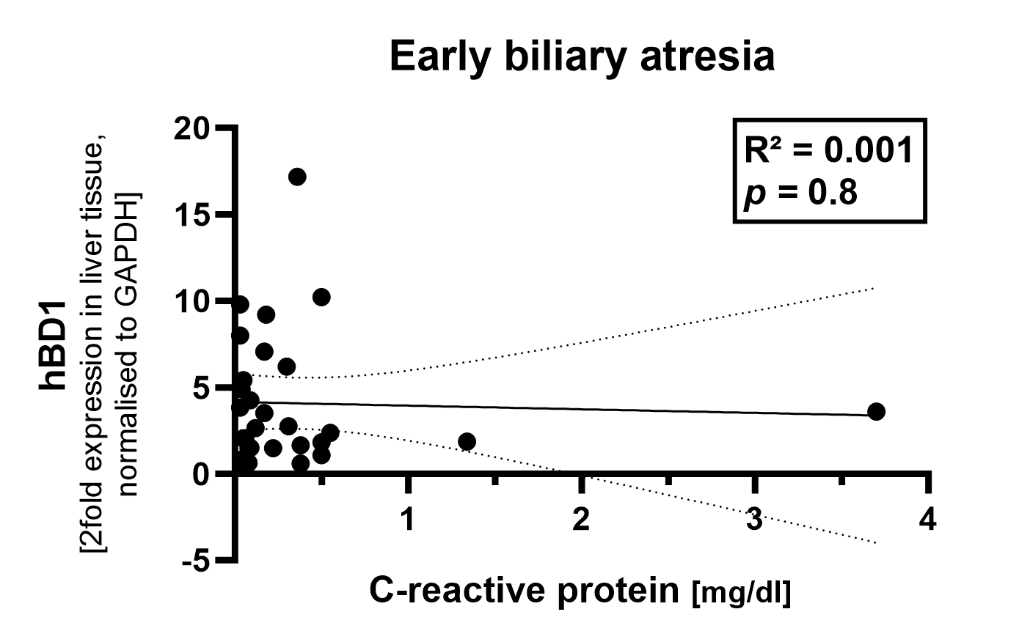


**Figure S3.** Correlation of *hBD1* liver expression with blood CRP. Linear regression analysis of liver expression of *hBD1* and blood CRP in eBA. Two-fold *hBD1* levels normalized to GAPDH versus CRP at biopsy date are indicated. R² and p values are shown. Abbreviations: CRP, C-reactive protein; eBA, early biliary atresia.

**References**

1. Bessho K, Mourya R, Shivakumar P, Walters S, Magee JC, Rao M, Jegga AG, Bezerra JA 2014 Gene expression signature for biliary atresia and a role for interleukin-8 in pathogenesis of experimental disease. Hepatology 60:211–223.
